# The relationship between adolescents’ physical activity, circadian rhythms, and sleep

**DOI:** 10.3389/fpsyt.2024.1415985

**Published:** 2024-08-14

**Authors:** Daoli Liu, Jin He, Hengfen Li

**Affiliations:** Department of Psychiatry, The First Affiliated Hospital of Zhengzhou University, Zhengzhou, Henan, China

**Keywords:** teenagers, sleep quality, circadian rhythm type, exercise, anxiety, depression

## Abstract

**Objective:**

To explore the correlation between physical activity, circadian rhythms, and sleep in adolescents, and analyze the influencing factors of sleep quality in this population.

**Methods:**

A total of 381 high school students were selected through cluster sampling in a specific high school. The Pittsburgh Sleep Quality Index (PSQI) was employed to categorize the participants into a good sleep quality group (n=199) and a poor sleep quality group (n=182). Comprehensive assessments were conducted using the Hamilton Depression Rating Scale (HAMD), Hamilton Anxiety Rating Scale (HAMA), PSQI, Morningness-Eveningness Questionnaire-5 (MEQ-5), and International Physical Activity Questionnaire Short Form (IPAQ-SF).

**Results:**

The prevalence of poor sleep quality is 47.8%. The BMI in the poor sleep quality group is higher than that in the good sleep quality group, and the male ratio is lower than that in the good sleep quality group. The poor sleep quality group exhibits significantly higher levels of depression, anxiety, evening chronotype, and low physical activity compared to the good sleep quality group.Spearman rank correlation analysis revealed a positive correlation between the PSQI total score and HAMA and HAMD scores, and a negative correlation with physical activity and MEQ-5 scores. Binary logistic stepwise regression analysis identified lack of physical activity, eveningness chronotype, anxiety, and depression as risk factors for poor sleep quality.

**Conclusion:**

Adolescent sleep quality is correlated with gender, BMI, anxiety, depression, chronotype, and physical activity levels. The findings highlight the importance of considering these factors in interventions aimed at improving sleep health in adolescents.

## 1 Introduction

Sleep, as a crucial physiological process, exerts a profound impact on the overall functioning of the human body (1). However, the prevalence of sleep disorders in the population is remarkably high, significantly affecting both the quality and duration of sleep and closely correlating with the incidence of various diseases. Firstly, there is a significant relationship between emotional well-being, depression risk, and sleep duration. Studies indicate that reports of poor mood, emotional regulation, and self-harm increase with insufficient sleep (2, 3). The relationship between emotions and sleep is complex and bidirectional, as low mood and anxiety can exacerbate insomnia, and vice versa (4). Research also suggests that extending sleep duration contributes to improving depressive symptoms. Secondly, sleep disorders are closely associated with an increased incidence of diseases in the digestive and circulatory systems (5). In general, sleep plays a crucial role in both mental and physical health.

Studies suggest that adolescents should aim for an average sleep duration of 8 to 10 hours per night to maintain optimal health (6). However, insomnia issues are prevalent among teenagers. Research by Dağ B et al. found that the prevalence of poor sleep quality among adolescents is approximately 40% (7), making insufficient sleep a serious concern in this age group. A nationwide survey in the United States reported that 72.7% of adolescents have an average nightly sleep duration of less than 8 hours (8), and this sleep deprivation increases the likelihood of engaging in risky behaviors, smoking, alcohol consumption, and aggressive behavior (9). At the onset of adolescence, due to biological adjustments in sleep duration, teenagers tend to adopt later circadian rhythms (10), making it challenging for them to align their sleep schedules with societal demands such as school, resulting in sleep deprivation (11). With the development of society, the increased use of electronic devices and academic pressures have become additional factors affecting adolescent sleep, contributing to a decline in sleep quality (12, 13).

Physical activity is defined as “any bodily movement produced by skeletal muscles that results in energy expenditure” (14). Lack of physical activity is a risk factor for various diseases, with research indicating that individuals who exercise regularly have a lower risk of coronary heart disease, diabetes, hypertension, and joint disorders compared to those who do not exercise regularly (15). However, in Chinese adolescents, engaging in physical activity faces numerous obstacles, such as a lack of time, inadequate facilities, and a lack of parental support. Despite these challenges, numerous studies have demonstrated a close relationship between insufficient physical activity and poor sleep quality.

Since previous research has paid limited attention to the relationship between physical activity, circadian rhythms, and sleep among Chinese adolescents, this study aims to assess these associations and provide a scientific basis for the health management of this specific population. In Chinese adolescents, it is crucial not only to determine their participation in physical activity but also to delve into the frequency, duration, and specific impact of these activities on sleep quality. Through this study, we can gain a more comprehensive understanding of the lifestyle of adolescents and offer robust support for the development of more effective health promotion measures

## 2 Objects and methods

### 2.1 Objects

Between September 16, 2023, and October 24, we employed a cluster sampling and cross-sectional survey method to select two classes each from the first, second, and third grades, as well as four classes from the first year of high school in a certain middle school in Henan. A total of 412 students were chosen as the study subjects. Inclusion criteria for the study were: ① Age between 13 and 17 years old; ② Han ethnicity; ③ Consent to participate in the survey. Exclusion criteria included: ① Those with concurrent physical illnesses; ② Those with a history of or current addiction to psychoactive substances. Ultimately, 31 students with concurrent physical illnesses or incomplete data were excluded, and the final study sample comprised 381 students.

The survey was conducted by 10 trained psychiatrists who distributed questionnaires and answered questions during the students’ response process. The survey was conducted in batches based on students’ study times, with each survey involving approximately 40 students from a single class. In total, 10 questionnaire surveys were conducted. A total of 412 questionnaires were distributed, with 381 valid questionnaires returned, achieving a response rate of 92.5%.

During each session of the questionnaire assessments, 10 psychiatrists and volunteers provided guidance and answered questions throughout. The Pittsburgh Sleep Quality Index (PSQI) [19], consisting of 19 self-reported questions, with a total score ranging from 0 to 21, was used. In this study, a score of ≥5 was defined as poor sleep quality. According to this criterion, 381 middle school students were classified as having good sleep quality (n=199) or poor sleep quality (n=182). The study obtained approval from the Ethics Committee of the First Affiliated Hospital of Zhengzhou University (Ethics Review Number: 2023-KY-0220–001). Prior to the survey, participants signed informed consent forms, and approval was obtained from relevant school authorities.

### 2.2 Tools

#### 2.2.1 Self-Developed General Questionnaire

① This includes demographic characteristics: gender, age, years of education, only child status, personality, parents’ personalities, and body mass index (BMI). ② General clinical features: including childhood trauma history and family history, with a total of 2 variables.

#### 2.2.2 International Physical Activity Questionnaire Short Version 

It is used to measure physical activity in the past week (16), comprising 7 items, including high-intensity and moderate-intensity exercise, walking, and sitting. Each type of physical activity is assigned an intensity code in metabolic equivalent of task (MET) units. The MET values are 3.3 for walking, 4.0 for moderate-intensity exercise, and 8.0 for high-intensity exercise. Total energy expenditure is estimated by multiplying the MET score by the minutes spent on each activity per week, expressed in MET-minutes/week. Physical activity levels are categorized as low, moderate, and high, with moderate and high levels combined as the physical activity group, and low level as the non-physical activity group.

#### 2.2.3 Hamilton Depression Rating-Scale-24-item version

This scale assesses the severity of depressive symptoms (17), with 24 items. The total score ranges from 0 to 74, with scores ≤8 indicating the absence of depressive symptoms, 9–19 indicating possible depressive symptoms, ≥20 indicating mild or moderate depression, and ≥35 indicating severe depressive symptoms. In this study, a total HAMD score ≥20 was considered indicative of depression.

#### 2.2.4 Hamilton Anxiety Scale

It is used to assess the severity of anxiety symptoms (18), with 14 items. The total score is 56, with ≤7 indicating the absence of anxiety symptoms, 8–13 indicating possible anxiety symptoms, ≥14 indicating mild anxiety, ≥21 indicating severe anxiety, and ≥29 indicating extremely severe anxiety. In the study, a total HAMA score ≥14 was considered indicative of anxiety.

#### 2.2.5 Pittsburgh Sleep Quality Index

This index is used for self-assessment of sleep disorders (19), including 19 self-reported questions and 5 questions rated by bed partners or roommates. In this study, only the self-reported questions were utilized. These questions contribute to scores in 7 components covering subjective sleep quality, sleep onset latency, sleep duration, habitual sleep efficiency, sleep disturbances, use of sleep medications, and daytime dysfunction. Each of these factors reflects the severity of the corresponding issue (0=none, 1=mild, 2=moderate, 3=severe), constituting an ordered categorical variable. The total score is the sum of these 7 factor scores, ranging from 0 to 21. Specifically, scores of 0–5 are classified as good sleep, 6–10 as average sleep, 11–15 as poor sleep, and ≥16 indicates very poor sleep quality. In this study, a score of ≥5 was defined as poor sleep quality.

#### 2.2.6 Morning and Evening Questionnaire 5

This questionnaire is used to assess students’ circadian rhythm types (20), comprising 5 items with a total score of 25. Based on the score, ≤11 is classified as evening type, and ≥12 as non-evening type.

### 2.3 Statistical methods

This study utilized SPSS 25.0 software for data analysis. For categorical data, frequencies (n) and percentages (%) were used for representation. For metric data conforming to a normal distribution, means ± standard deviations were used for representation, while for skewed metric data, the representation used was (median, interquartile range/between the upper and lower quartiles). In comparing the demographic data, general clinical features, and the composition ratios of HAMD, HAMA, and MEQ-5 scales between the groups with good and poor sleep quality, the chi-square (χ²) test was employed. For metric data such as BMI, years of education, and age, as well as comparisons of ordered multiclass variables such as physical activity levels (low, moderate, high), subjective sleep quality, sleep onset latency, sleep duration, habitual sleep efficiency, sleep disturbances, use of sleep medications, and daytime dysfunction, the Mann-Whitney U test was used, and comparisons among three groups were conducted using the Kruskal-Wallis test. Additionally, Spearman rank correlation analysis and binary logistic stepwise regression analysis (1=good sleep quality, 2=poor sleep quality) were conducted to explore the factors related to sleep quality. In all statistical analyses, the significance level was set at P<0.05, indicating statistical significance for observed differences.

## 3 Result

### 3.1 Comparison of general demographic data

#### 3.1.1 Comparison of general demographic data between good sleep quality group and poor sleep quality group

Among the study participants, there were 199 individuals in the good sleep quality group (52.2%) and 182 individuals in the poor sleep quality group (47.8%). The comparison of general demographic data between the two groups revealed that the poor sleep quality group had a higher BMI compared to the good sleep quality group, and the proportion of males in the poor sleep quality group was lower than that in the good sleep quality group (**Table 1**).

**Table 1:** Comparison of general demographic data between two groups [M (P25, P75)/Number (%)] (n=381).

Variable		Good Sleep Quality	Poor Sleep Quality	U Value/χ² Value	P Value
		n=199	n=182		
Age (years) M(P25,P75)		14.0(13.0,15.0)	14.0(14.0,15.0)	17444.50u	0.523
Years of Education M(P25,P75)		9.0(8.0,10.0)	9.0(8.0,10.0)	18048.50u	0.953
BMI(kg/m²) M(P25,P75)		18.8(17.3,23.5)	19.7(18.0,27.2)	15172.00u	0.006*
Gender (Male) (%)		136(68.3)	100(54.9)	7.24a	0.008*
Only Child/yes(%)		5(2.5)	8(4.4)	1.02a	0.401
Personality(%)	Introverted	43(21.6)	51(28.0)	2.17a	0.337
	General	114(57.3)	94(51.6)		
	Extroverted	42(21.1)	37(20.3)		
Father’s Personality (%)	Introverted	6(3.0)	7(3.8)	1.31a	0.524
	General	142(71.4)	137(75.3)		
	Extroverted	51(25.6)	38(20.9)		
Mother’s Personality (%)	Introverted	6(3.0)	4(2.2)	0.55f	0.781
	General	151(75.9)	135(74.2)		
	Extroverted	42(21.1)	43(23.6)		
Childhood Trauma History	Yes	13(6.5)	19(10.4)	1.89a	0.197
	No	186(93.5)	163(89.6)		
Family History	Yes	6(3.0)	12(6.6)	2.70a	0.146
	No	193(97.0)	170(93.4)		

M represents mean, P25 represents the 25th percentile, P75 represents the 75th percentile, Count represents the count of individuals, and % represents the percentage. U value is for Mann-Whitney U test, and χ² value is for the chi-square test. * indicates statistical significance (P < 0.05).

#### 3.1.2 Comparison of general demographic data between evening type and non-evening type groups

Among the study participants, there were 140 individuals in the evening type group (36.7%) and 241 individuals in the non-evening type group (63.3%). The results of the comparison of general demographic data between the two groups showed no significant differences (**Table 2**).

**Table 2:** Comparison of general demographic data between two groups [M (P25, P75)/Count (%)] (n=381).

Variable		Evening Type	Non-Evening Type	U Value/χ² Value	PValue
		n=140	n=241		
Age (years) M(P25,P75)		15.0(14.0,15.0)	14.0(13.0,15.0)	15332.50u	0.125
Years of Education M(P25,P75)		9.0(8.0,10.0)	9.0(8.0,10.0)	14957.00u	0.056
BMI(kg/m²) M(P25,P75)		19.3(17.8,24.6)	19.3(17.5,23.7)	16480.00u	0.705
Gender (Male) (%)		85(60.7)	151(62.7)	0.14a	0.743
Only Child/yes(%)		5(3.6)	8(3.3)	0.02a	0.896
Personality(%)	Introverted	33(23.6)	51(25.3)	3.37a	0.190
	General	71(50.7)	94(56.8)		
	Extroverted	36(25.7)	37(17.8)		
Father’s Personality (%)	Introverted	4(2.9)	9(3.7)	0.257a	0.887
	General	104(74.3)	175(72.6)		
	Extroverted	32(22.9)	57(23.7)		
Mother’s Personality (%)	Introverted	6(4.3)	4(1.7)	5.34f	0.063
	General	110(78.6)	176(73.0)		
	Extroverted	24(17.1)	61(25.3)		
Childhood Trauma History	Yes	17(12.1)	15(6.2)	4.03a	0.055
	No	123(87.9)	226(93.8)		
Family History	Yes	10(7.1)	8(3.3)	2.88a	0.090
	No	130(92.9)	233(96.7)		

Same as **Table 1**.

### 3.2 Comparison of depression, anxiety, chronotype, and physical activity levels between two groups

The comparison of the composition ratios of depression, anxiety, chronotype, and physical activity levels between the two groups revealed statistically significant differences. The poor sleep quality group exhibited higher rates of depression and anxiety compared to the good sleep quality group. The poor sleep quality group also showed a higher proportion of individuals with an evening chronotype but a lower proportion with a morning chronotype compared to the good sleep quality group. Additionally, the poor sleep quality group had a lower proportion of individuals with high physical activity levels compared to the good sleep quality group.

In terms of the comparison of total scores on depression, anxiety, sleep quality, chronotype, and physical activity levels between the two groups, there were statistically significant differences. The overall distribution of HAMA and HAMD total scores in the poor sleep quality group was higher than that in the good sleep quality group, while the overall distribution of MEQ-5 total scores was lower in the poor sleep quality group than in the good sleep quality group. Using Wilcoxon rank-sum tests with physical activity levels (low=1, moderate=2, high=3) as the independent variable, the overall distribution of physical activity levels in the good sleep quality group was found to be greater than that in the poor sleep quality group (**Table 3**).

**Table 3:** Comparison of the composition ratio and total scores of depression, anxiety, chronotype, and physical activity level between two groups [M (P25, P75)/Number (Percentage)].

Variable		Good Sleep Quality	Poor Sleep Quality	χ² Value/U Value	PValue
		n=199	n=182		
HAMD	≥20(%)	20(10.1)	68(37.2)	39.92a	<0.001
	<20(%)	179(89.9)	114(62.6)		
	Total Score, M(P25,P75)	5.0(1.0,11.0)	16.0(8.0,26.0)	8954.00u	<0.001
HAMA	≥14(%)	25(12.6)	83(45.6)	51.10a	<0.001
	<14(%)	174(87.4)	99(54.4)		
	Total ScoreM(P25,P75)	5.0(2.0,10.0)	12.0(6.0,20.0)	9504.00u	<0.001
MEQ-5	Evening Type (%)	54(27.1)	86(47.3)	16.55a	<0.001
	Non-Evening Type (%)	145(72.9)	96(52.7)		
	Total ScoreM(P25,P75)	15.0(11.0,18.0)	12.0(10.0,16.0)	12452.50u	<0.001
IPAQ-SF	Low Physical Activity (%)	56(27.1)	67(36.8)	10.03a	0.006
	Low Physical Activity (%)	41(20.6)	51(28.0)		
	High Physical Activity (%)	102(51.3)	64(35.2)		
	M(P25,P75)	3.0(1.0,3.0)	2.0(1.0,3.0)	15249.50h	0.004

HAMD, Hamilton Depression Rating Scale 24-item version; HAMA, Hamilton Anxiety Scale; MEQ-5, Morning and Evening Questionnaire 5; IPAQ-SF, International Physical Activity Questionnaire Short Version. a: χ² test, u: Mann-Whitney U test, h: Kruskal-Wallis test.

### 3.3 Comparison of physical activity level and chronotype with PSQI and seven factor scores

Regarding sleep duration, among the 381 participants, 196 (51.4%) reported sleeping for more than 7 hours, 118 (30.9%) slept between 6 to 7 hours, 39 (10.2%) had a sleep duration of 5 to 6 hours, and 28 (7.3%) reported sleeping less than 5 hours.

#### 3.3.1 Comparison of different physical activity levels with PSQI and seven factor scores (subjective sleep quality, sleep onset latency, sleep duration, habitual sleep efficiency, sleep disturbances, use of hypnotics, and daytime dysfunction)

The distribution of PSQI total scores in the high physical activity level group was lower than those in the moderate and low physical activity level groups. No significant differences were observed in the comparison of the seven factor scores among the three groups (**Table 4**).

**Table 4:** Comparison of PSQI seven factor scores among different physical activity levels.

Variable	Low Physical Activity Level	Moderate Physical Activity Level	High Physical Activity Level	H Value	P Value
	n=123	n=92	n=166		
	M(P25,P75)	M(P25,P75)	M(P25,P75)		
Subjective Sleep Quality	1(1,1)	1(1,1.5)	1(1, 1)	5.38	0.065
Sleep Onset Time,	1(1,2)	1(0,2)	1(0,2)	3.71	0.157
Sleep Duration	1(0,1)	1(0, 1)	0(0,1)	4.22	0.121
Sleep Efficiency	0(0,1)	0(0, 1)	0(0,0)	4.33	0.115
Sleep Disturbances	1(1, 2)	1(1, 2)	1(1, 2)	0.88	0.645
Use of Sleep Medications	0(0,0)	0(0,0)	0(0,0)	0.54	0.763
Daytime Dysfunction	1(0,1)	1(1,1)	1(0,1)	4.63	0.097
PSQI Total Score	6(4,8)	6(3,9)	5(3,7)	7.74	0.022

H Value: Kruskal-Wallis Test.

#### 3.3.2 Comparison of circadian rhythm types with PSQI and seven factor scores

The distribution of subjective sleep quality, sleep onset time, sleep duration, habitual sleep efficiency, sleep disturbances, daytime functioning, and PSQI total score for evening types is higher than that of non-evening types (**Table 5**).

**Table 5:** Comparison of circadian rhythm types and PSQI seven factor scores.

Variable	Evening Type	Non-Evening Type	U Value/χ² Value	PValue
	n=140	n=241		
	M(P25,P75)a	M(P25,P75)		
Subjective Sleep Quality	1(1,2)	1(1,1)	13997.50	0.001
Sleep Onset Time,	1(0.5,2)	1(0,2)	14324.00	0.011
Sleep Duration	1(0,2)	0(0, 1)	13012.50	<0.001
Sleep Efficiency	0(0,1)	0(0, 0)	14898.00	0.016
Sleep Disturbances	1(1, 2)	1(1, 1)	14844.50	0.029
Use of Sleep Medications	0(0,0)	0(0,0)	15940.00	0.077
Daytime Dysfunction	1(0,1)	1(0,1)	14479.50	0.011
PSQI Total Score	6(4,9)	5(3,7)	12049.00	<0.001

u value Mann-Whitney U test.

### 3.4 Relationship between PSQI total score and anxiety, depression, physical activity,and circadian rhythm in adolescents

Spearman Rank Correlation analysis revealed a positive correlation between the total PSQI score in adolescents and HAMA and HAMD scores, and a negative correlation with physical activity level and MEQ-5 scores (**Table 6**).

**Table 6:** Correlation coefficients between PSQI score and physical activity level, anxiety, depression, and circadian rhythm.

	correlation coefficients	Physical Activity Level	HAMA	HAMD	MEQ-5
PSQI	r	-0.121*	0.497**	0.537**	-0.316**

MEQ-5, Morningness-Eveningness Questionnaire; HAMD, Hamilton Depression Rating Scale; HAMA, Hamilton Anxiety Rating Scale; PSQI, Pittsburgh Sleep Quality Index. * p < 0.05, ** p < 0.01.

### 3.5 Relationship between physical activity, circadian rhythm, and the seven factors of PSQI in adolescents

Spearman rank correlation analysis revealed no significant linear correlation between physical activity levels and the seven factors of PSQI (**Table 7**). Spearman rank correlation analysis revealed a negative correlation between MEQ-5 scores and subjective sleep quality, sleep onset time, sleep duration, habitual sleep efficiency, sleep disturbances, and daytime functioning (**Table 8**).

**Table 7:** Correlation coefficients between physical activity levels and the seven factors of PSQI.

		Subjective Sleep Quality	Sleep Onset Time	Sleep Duration	Sleep Efficiency	Sleep Disturbances	Use of Sleep Medications	Daytime Dysfunction
Physical Activity Level	r	-0.096	-0.090	-0.090	-0.087	-0.015	0.010	0.014
	P	0.060	0.078	0.079	0.089	0.773	0.842	0.785

**Table 8:** The correlation coefficients between MEQ-5 scores and the seven factors of PSQI.

		Subjective Sleep Quality	Sleep Onset Time	Sleep Duration	Sleep Efficiency	Sleep Disturbances	Use of Sleep Medications	Daytime Dysfunction
MEQ-5 scores	r	-0.202**	-0.162**	-0.204**	-0.16**	-0.138**	-0.098	-0.121*

“MEQ-5 refers to the Morningness-Eveningness Questionnaire. *P < 0.05, **P < 0.01”.

### 3.6 Factors influencing sleep quality in adolescents

“In logistic stepwise regression analysis with sleep quality (1=good, 2=poor) as the dependent variable, and anxiety (1=none, 2=present), depression (1=none, 2=present), circadian rhythm (1=evening type, 2=non-evening type), physical activity level (1=no physical activity, 2=physical activity present - moderate to high level), and gender (1=male, 2=female) as independent variables, the results revealed that no physical activity, evening type, anxiety, and depression were identified as risk factors for sleep quality (see **Table 9**)”.

**Table 9:** Risk factors influencing sleep quality in adolescents.

Variable	B value	Standard Error	OR (95% CI)	P value
Depressive	0.90	0.33	2.45(1.29~4.45)	0.006
Anxiety	1.39	0.31	4.02(2.21~7.30)	<0.001
Physical Activity	-0.52	0.25	0.59(0.36~0.97)	0.037
Evening Type	0.57	0.24	1.77(1.10~2.85)	0.018

## 4 Discussion

This study found that the prevalence of poor sleep quality among adolescents was 47.8%, with results from other countries ranging between 28.2% and 76.5% (7, 21–24). In Chinese studies, the occurrence of poor sleep quality was reported to be 26.9% (25). These variations suggest a wide disparity in adolescent sleep quality between domestic and international research, possibly influenced by differences in participant sources.

Additionally, our study revealed that the BMI of the poor sleep quality group was higher than that of the good sleep quality group, aligning with findings from international studies (26). The percentage of females in the poor sleep quality group was also higher, consistent with foreign research findings (27). Furthermore, the poor sleep quality group exhibited higher rates of anxiety, depression, and evening chronotype, consistent with previous studies (4, 11). In terms of physical activity level, the overall activity level of the poor sleep quality group was lower, resembling findings in studies among college students (28).Regarding sleep duration, our investigation showed that 48.6% of adolescents had a sleep duration of less than 7 hours. In comparison, Chinese research reported a sleep insufficiency rate (less than 8 hours) of 50.9% among adolescents (29), while a U.S. survey indicated that 72.7% of adolescents did not get an average of 8 hours of sleep on school nights (8). This suggests that both domestic and international adolescents face significant.

Spearman rank correlation analysis revealed that the PSQI total score in adolescents is positively correlated with HAMA scores and HAMD scores, and negatively correlated with physical activity level and MEQ-5 scores. This suggests that adolescents with emotional issues, an evening chronotype, and lower physical activity levels are more likely to experience sleep quality problems. Additionally, the analysis found negative correlations between MEQ-5 scores and subjective sleep quality, sleep onset time, sleep duration, habitual sleep efficiency, sleep disturbances, and daytime functioning. This indicates that adolescents with an evening chronotype are more prone to various sleep-related issues. In binary logistic stepwise regression analysis, after controlling for gender, no physical activity, evening chronotype, anxiety, and depression remained identified as risk factors for sleep quality. This study revealed that almost half of adolescents experience poor sleep quality. Those who engage in regular physical activity have better sleep quality compared to their less active counterparts. Adolescents are in a critical stage of the second peak of growth and development, and having good, sufficient sleep is crucial for their mental health. Therefore, it is necessary to strengthen sleep hygiene education, starting with improving school schedules and increasing physical activity time, to promote sleep health among primary and secondary school students. Guiding and encouraging adolescents to establish a scientific and feasible daily routine is of great significance in preventing the occurrence of sleep problems.

This study is a cross-sectional investigation into adolescent sleep and its related factors; however, it lacks long-term follow-up surveys. The surveyed individuals are all students in a particular school, posing limitations to the representativeness of the findings. Due to the potential variability of physical activity behavior over an academic year (e.g., around exams or at the beginning or end of holidays), there may be measurement errors in the assessment of physical activity levels. Additionally, the study relies on self-reported data from students rather than employing more objective methods. Finally, the study is limited to one school, resulting in insufficient representativeness. To better understand the changes in adolescent sleep patterns, further longitudinal studies are necessary, allowing for the identification of more specific school management improvements. The limitations of the study underscore the importance of ongoing research to inform targeted interventions and refine school-based strategies for promoting healthy sleep habits among adolescents.

## Data Availability

The original contributions presented in the study are included in the article/supplementary material. Further inquiries can be directed to the corresponding author.
